# The Evaluation of Type 2 Diabetes Mellitus Related Changes in Diastolic Dysfunction During Exercise Using Conventional and Tissue Doppler Echocardiography

**DOI:** 10.14740/cr439w

**Published:** 2015-12-16

**Authors:** Hakan Ozkan, Sedat Akdemir, Selma Tiryakioglu, Hasan Ari, Tahsin Bozat

**Affiliations:** a Bahcesehir University, Faculty of Medicine, Department of Cardiology, Istanbul, Turkey; b Bursa Yuksek Ihtisas Hospital, Department of Cardiology, Bursa, Turkey; c Bursa State Hospital, Department of Cardiology, Bursa, Turkey

**Keywords:** Diastolic dysfunction, Type 2 diabetes mellitus, Impaired exercise capacity, Tissue Doppler

## Abstract

**Background:**

The aim of this study was to evaluate the relationship between changes in diastolic functions during exercise and the exercising capacity in diabetic patients with diastolic dysfunction and to compare them with healthy individuals and diabetic patients without diastolic dysfunction.

**Methods:**

Totally 70 patients prospectively were included in the study and three groups were formed. Forty-six diabetic patients were divided into two groups: those with (group 1) and without (group 2) diastolic dysfunction. The control group (group 3) consisted of 24 patients. All patients were subjected to treadmill exercising test. Echocardiographical assessment was made before exercise and immediately after peak exercise.

**Results:**

Exercising time was dramatically decreased in group 1 compared to the other groups (group 1: 396 ± 125 second, group 2: 487 ± 66 second and group 3: 519 ± 102 second). In group 1, the diastolic mitral flow pattern at rest was transformed into pseudo-normal pattern at peak exercise from abnormal relaxation pattern (E/A ratio 0.70 ± 0.11 during rest, 1.02 ± 0.16; P < 0.0001 during peak exercise). Deceleration time (DT) and iso-volumetric relaxation time (IVRT) turned to normal values (DT 238.86 ± 39.48 millisecond during rest and 199.5 ± 23.57 millisecond during peak exercise; P = 0.001, IVRT 102.83 ± 16.22 millisecond during rest and 74.36 ± 8.67 millisecond during peak exercise; P = 0.001). In groups 2 and 3, the mitral flow pattern, DT and IVRT remained within normal limits during rest and exercise. E/Em ratio, which is one of the parameters of tissue Doppler, increased during peak exercise in the diabetic group with diastolic dysfunction (E/Em ratio 7.85 ± 3.31 during rest and 11.14 ± 3.40 after peak exercise; P < 0.0001).

**Conclusions:**

Diabetic patients with diastolic dysfunction demonstrated a reduced exercise capacity, which may be due to aggravation of pre-existing left ventricular dysfunction.

## Introduction

Diastolic functions during exercise may be used as an early sign of this diabetic heart disease preceding the systolic damage due to its independent association with exercise capacity. To evaluate the relationship between changes in diastolic functions during exercise and the exercising capacity in diabetic patients with diastolic dysfunction who have not a previous history of cardiovascular disease may reveal early sings of diabetic cardiac disease. Mitral annulus pulse-wave tissue Doppler (PWTD) imaging is the relatively new approach for the assessment of left ventricular diastolic functions.

## Material and Methods

A total of 70 subjects (51 men, 19 women, mean age 52.8 ± 4.2 years) are composed of type 2 diabetes mellitus patients and normal individuals who applied to the cardiology department. Diabetic patients suffering from exercitional dyspnea referred from internal medicine policlinic for further evaluation were prospectively enrolled in the study. Those who have a previous history of coronary artery disease, hypertension, peripheral artery disease, cerebrovascular disease, echocardiographically diagnosed heart valve disease, left ventricle hypertrophy and/or a segmental contractility defect, any ST-T change in ECG, chest pain and/or ischemia detected by treadmill exercise test were not included in the study.

Diabetic patients were divided into two groups according to echocardiographic evidence of diastolic dysfunction. Group 1 consisted of 30 diabetic patients with diastolic dysfunction and group 2 included 16 diabetic patients without diastolic dysfunction. The control group (group 3) consisted of 24 healthy volunteers. Assessment was made between three groups. The following criteria were used for the diagnosis of diastolic dysfunction: 1) E/A ratio < 1 or > 2; 2) deceleration time (DT) < 150 or > 220 ms; 3) iso-volumetric relaxation time (IVRT) < 60 or > 100 ms; 4) annular tissue Doppler velocity in any section < 8 cm/s. All patients in group 1 met E/A ratio < 1, DT > 220 and IVRT > 100 criteria for diagnosis of stage 1 diastolic dysfunction.

All patients underwent physical examinations after taking detailed anamnesis. Their body mass indices (kg/m^2^) were calculated. Using the venous blood samples of diabetic patients, fasting blood glucose levels and HbA1c levels were checked. M-mode and Doppler echocardiographical parameters were assessed at rest in all patients. All patients underwent exercising test using Bruce protocol. Exercise capacity was assessed by both exercise duration and metabolic equivalent (MET). Immediately after cessation of maximal exercise, left ventricular diastolic functions were reassessed by Doppler echocardiography.

Transthoracic echocardiographies of subjects were made using 3.5 - 4.5 MHz probe with Doppler echocardiography device (Vivid 7 Pro GE) in left lateral decubitus position. Echocardiographical parameters were measured from at least three consecutive cardiac cycles during expirium and the mean values were calculated. M-mode tracings from the parasternal long-axis view were used to measure left atrium size, left ventricular wall thickness and diameters, ejection fraction and fractional shortening as the systolic function indicators. Furthermore left ventricular mass (LVM) was calculated by using the formula: LVM = 0.8 × 1.04((Left ventricular end-diastolic diameter + septum thickness + posterior wall thickness)^3^ - (Left ventricular end-diastolic diameter)^3^) + 0.6) [1].

Pulsed-wave (PW) Doppler is performed in the apical four-chamber view at the level of mitral valve leaflet tips to measure early diastolic flow peak velocity (E), late diastolic flow peak velocity (A), early and late diastolic flow peak velocity ratio (E/A), E wave DT (the interval from the peak of E to the point where the deceleration curve lowers to the baseline) and IVRT (the period between when blood flow through the aortic valve ceases and when it begins through the mitral valve) respectively. Pulmonary vein flow velocities were assessed from apical four-chamber view by placing PW sample volume 0.5 - 1 cm upstream in the right upper pulmonary vein. Pulmonary vein systolic flow peak velocity (S), pulmonary vein diastolic flow peak velocity (D), pulmonary vein reverse flow velocity (Ap) and S/D ratio were measured. PWTD sampling was performed at the level of the lateral mitral annulus. A PW Doppler sample volume at 1 cm within the lateral MV insertions of MV leaflets over an A4C view. All Doppler signals were recorded with a chart recorder set at 100 mm/s. The average of three end-expiratory cycles was used. Parallel to the long axis of the left ventricular wall early diastolic wave peak velocity (Em), late diastolic wave peak velocity (Am), the ratio of early and late diastolic wave peak velocities (Em/Am), and ratio of early mitral diastolic flow peak velocity to early mitral annular diastolic flow peak velocities (E/Em) were calculated respectively.

Patients with suspected coronary artery disease and those with insufficient exercise capacity underwent Tc-99m MIBI SPECT myocardial perfusion scintigraphy. Patients with myocardial perfusion defect were excluded from the study. Informed consent was given to all participants.

Statistical analysis of collected data was conducted using SPSS 8.0 Windows statistic package program. All hemodynamic and left ventricular diastolic function parameters were assessed within and among the groups at rest and after peak exercise. Parametric data were assessed using Student’s *t*-test and non-parametrical data were analyzed with Chi-square test. The distribution of the groups in terms of basic features was assessed using Kruskal-Wallis test. A value of P < 0.05 was considered statistically significant.

## Results

The mean age of 51 female and 19 male patients who participated in the study was 52.8 ± 4.2 years (range 35 - 60 years). Baseline characteristics of the three groups are shown in Table 1.

**Table 1:** Baseline Characteristics of the Patients

Parameters	Group 1 (n = 30)	Group 2 (n = 16)	Group 3 (n = 24)	P value		
				1-2	1-3	2-3
Age (years)	53.6 ± 4.5	52.1 ± 5.2	52.3 ± 3.4	NS	NS	NS
Sex (n, %)						
Male	6 (20%)	6 (-37.5%)	7 (29.1%)	NS	NS	NS
Female	24 (80%)	10 (%62,5)	17 (70.9%)	NS	NS	NS
Treatment						
Oral hypoglycemic agent	25	13		NS		
Insulin	3	2		NS		
Diet	2	1		NS		
Diabetes duration (years)	4.8 ± 4.2	3.4 ± 2.6		NS		
FBG (mg/dL)	158.6 ± 34.3	152.1 ± 31		NS		
HbA1c (%)	8.1 ± 1.7	7.1 ± 1.5		NS		
BMI (kg/m^2^)	27.9 ± 2.5	28.4 ± 2.4	27.9 ± 1.9	NS	NS	NS

FBG: fasting blood glucose; HbA1c: hemoglobin A1c; BMI: body mass index; NS: non-significant.

There were not any significant differences among groups in M-mode transthoracic echocardiographic measurements (Table 2).

**Table 2:** M-Mode Echocardiographical Measurements of the Groups

Parameters	Group 1	Group 2	Group 3	P value		
				1-2	1-3	2-3
Left ventricle end-diastolic diameter (cm)	4.59 ± 0.89	4.41 ± 0.41	4.66 ± 0.38	NS	NS	NS
Left ventricle end-systolic diameter (cm)	2.83 ± 0.64	2.76 ± 0.38	2.78 ± 0.36	NS	NS	NS
Septum thickness (cm)	0.96 ± 0.11	0.99 ± 0.09	0.92 ± 0.11	NS	NS	NS
Posterior wall thickness (cm)	0.88 ± 0.12	0.91 ± 0.12	0.84 ± 0.12	NS	NS	NS
Left atrium (cm)	3.77 ± 0.29	3.60 ± 0.23	3.60 ± 0.32	NS	NS	NS
Fractional shortening (%)	38 ± 7.27	36.8 ± 5.68	40.35 ± 6.29	NS	NS	NS
Ejection fraction (%)	69 ± 8.09	66 ± 6.60	69.87 ± 6.22	NS	NS	NS
Left ventricle mass (g)	150.06 ± 20.09	142.4 ± 21.20	139.8 ± 27.40	NS	NS	NS

NS: non-significant.

In diabetic patients with diastolic dysfunction (group 1), significant increases in heart rates, systolic and diastolic blood pressures were detected at peak exercise when compared to the resting values. Peak early diastolic mitral flow velocity (E) was detected significantly increased, the peak late diastolic flow velocity (A) velocity was also increased but this increase was not statistically significant (E wave velocity 0.66 ± 0.14 m/s at rest, 0.99 ± 0.16 m/s after peak exercise; P < 0.0001, A wave velocity 0.93 ± 0.15 m/s at rest, 0.98 ± 0.21 m/s after peak exercise; P = 0.068). For that reason, the E/A ratio changed significantly and showed pseudo-normalization after peak exercise (E/A ratio 0.70 ± 0.11 in relaxation and 1.02 ± 0.16; P < 0.0001 after peak exercise). Comparison of group 1 hemodynamic and diastolic function parameters at rest and after peak exercise is summarized in Table 3, data of group 2 are summarized in Table 4 and data of group 3 are shown in Table 5. E/A ratio before and after exercise significantly changed however still remained > 1. Therefore it was not significant for diastolic dysfunction in group 2. Tissue Doppler findings showing diastolic functions parameters were not statistically significant.

**Table 3:** Comparison of Resting and Post-Exercise Hemodynamic and Diastolic Function Parameters in Group 1

Parameters	Pre-exercise	Post-exercise	P value
SBP (mm Hg)	116.66 ± 8.33	157.00 ± 18.22	0.001
DBP (mm Hg)	75.93 ± 8.02	88.5 ± 8.82	0.001
HR (pulse/min)	87.43 ± 9.42	148.4 ± 15.39	< 0.0001
E (m/s)	0.66 ± 0.14	0.99 ± 0.16	< 0.0001
A (m/s)	0.93 ± 0.15	0.98 ± 0.21	0.068
E/A	0.70 ± 0.11	1.02 ± 0.16	< 0.0001
DT (ms)	238.86 ± 39.48	199.5 ± 23.57	0.001
IVRT (ms)	102.83 ± 16.22	74.36 ± 8.67	0.001
S (m/s)	0.58 ± 0.12	0.65 ± 0.12	0.05
D (m/s)	0.54 ± 0.10	0.62 ± 0.15	0.04
S/D	1.07 ± 0.24	1.05 ± 0.28	NS
Ap (m/s)	0.35 ± 0.005	0.45 ± 0.08	0.002
Em (m/s)	0.09 ± 0.025	0.11 ± 0.78	NS
Am (m/s)	0.11 ± 0.078	0.14 ± 0.09	NS
Em/Am	0.79 ± 0.4	0.77 ± 0.23	NS
E/Em	7.85 ± 3.31	11.14 ± 3.40	< 0.0001

SBP: systolic blood pressure; DBP: diastolic blood pressure; HR: heart rate; DT: deceleration time; IVRT: iso-volumetric relaxation time; NS: non-significant.

**Table 4:** Comparison of Resting and Post-Exercise Hemodynamic and Diastolic Function Parameters in Group 2

Parameters	Pre-exercise	Post-exercise	P value
SBP (mm Hg)	113.12 ± 9.28	143.12 ± 13.52	< 0.0001
DBP (mm Hg)	73.75 ± 5.91	82.18 ± 3.63	0.001
HR (pulse/min)	82.87 ± 3.63	161.5 ± 10.99	< 0.0001
E (m/s)	0.89 ± 0.13	0.97 ± 0.16	NS
A (m/s)	0.77 ± 0.11	0.94 ± 0.17	0.01
E/A	1.12 ± 0.08	1.02 ± 0.13	0.006
DT (ms)	185.31 ± 21	193.87 ± 28.81	NS
IVRT (ms)	77.31 ± 8.93	70.75 ± 9.18	NS
S (m/s)	0.69 ± 0.093	0.78 ± 0.13	NS
D (m/s)	0.55 ± 0.10	0.68 ± 0.19	0.014
S/D	1.27 ± 0.19	1.20 ± 0.30	NS
Ap (m/s)	0.28 ± 0.02	0.34 ± 0.07	NS
Em (m/s)	0.12 ± 0.01	0.13 ± 0.01	0.02
Am (m/s)	0.10 ± 0.02	0.12 ± 0.02	0.03
Em/Am	1.0 ± 0.41	1.0 ± 0.33	NS
E/Em	7.3 ± 1.63	7.26 ± 1.41	NS

SBP: systolic blood pressure; DBP: diastolic blood pressure; HR: heart rate; DT: deceleration time; IVRT: iso-volumetric relaxation time; NS: non-significant.

**Table 5:** Comparison of Resting and Post-Exercise Hemodynamic and Diastolic Function Parameters in Group 3

Parameters	Pre-exercise	Post-exercise	P value
SBP (mm Hg)	114.37 ± 10.35	141.25 ± 13.61	< 0.0001
DBP (mm Hg)	78.16 ± 6.53	82.08 ± 4.87	0.02
HR (pulse/min)	81.37 ± 8.77	161.54 ± 16.55	< 0.0001
E (m/s)	0.93 ± 0.14	1.17 ± 0.16	< 0.0001
A (m/s)	0.70 ± 0.11	0.93 ± 0.16	< 0.0001
E/A	1.33 ± 0.22	1.25 ± 0.17	0.01
DT (ms)	172.2 ± 19.24	178.16 ± 21.25	NS
IVRT (ms)	76.29 ± 8.13	69.5 ± 7.42	0.007
S (m/s)	0.61 ± 0.083	0.84 ± 0.017	< 0.0001
D (m/s)	0.48 ± 0.057	0.65 ± 0.12	< 0.0001
S/D	1.24 ± 0.13	1.23 ± 0.27	NS
Ap (m/s)	0.26 ± 0.038	0.32 ± 0.05	0.002
Em (m/s)	0.14 ± 0.026	0.156 ± 0.034	0.006
Am (m/s)	0.10 ± 0.029	0.11 ± 0.026	NS
Em/Am	1.45 ± 0.53	1.44 ± 0.51	NS
E/Em	6.15 ± 1.58	7.72 ± 1.50	0.002

SBP: systolic blood pressure; DBP: diastolic blood pressure; HR: heart rate; DT: deceleration time; IVRT: iso-volumetric relaxation time; NS: non-significant.

No significant difference was observed among groups at rest in terms of heart rate (group 1: 87.43 ± 9.42, group 2: 82.87 ± 3.63, group 3: 81.37 ± 8.77; P value 1-2 = 0.38, P value 1-3 = 0.27, P value 2-3 = 0.98), systolic (group 1: 116.66 ± 8.33, group 2: 113.12 ± 9.28, group 3: 114.37 ± 10.35; P value 1-2 = 0.89, P value 1-3 = 0.71, P value 2-3 = 0.88) and diastolic blood pressures (group 1: 75.93 ± 8.02, group 2: 73.75 ± 5.91, group 3: 78.16 ± 6.53; P value 1-2 = 0.69, P value 1-3 = 0.57, P value 2-3 = 0.31). In both diabetic patient groups, the rate of E wave was lower (E wave velocity group 1: 0.66 ± 0.14 m/s, group 2: 0.89 ± 0.13 m/s, group 3: 0.93 ± 0.14 m/s), and the velocity of A wave was higher than control group (group 1: 0.93 ± 0.15 m/s, group 2: 0.77 ± 0.11 m/s, group 3: 0.70 ± 0.11 m/s). In group 1, E/A ratio was < 1 and DT and IVRT were longer than normal. In other two groups, these values were within normal limits. Assessment of pulmonary vein recordings showed significantly higher Ap wave velocity in group 1 than other groups (group 1: 0.35 ± 0.005 m/s, group 2: 0.28 ± 0.02 m/s, group 3: 0.26 ± 0.038 m/s). The lowest S/D ratio was also detected in group 1 but the difference was not statistically significant. Comparing the lateral mitral annulus measurements Em velocity (group 1: 0.09 ± 0.025 m/s, group 2: 0.12 ± 0.01 m/s, group 3: 0.14 ± 0.02 m/s) and Em/Am ratios (group 1: 0.79 ± 0.40; group 2: 1.0 ± 0.41; group 3: 1.45 ± 0.53 m/s) were detected significantly lower in group 1 than other groups. Immediately after the peak exercise in group 1 E/A ratio, DT and IVRT turned to normal ranges when S and D wave velocity and the S/D ratio were detected lower than that observed by two other groups. However, S/D ratio was not statistically significant between three groups (Table 6).

**Table 6:** Comparison of Post-Exercise Hemodynamic and Echocardiographical Doppler Parameters Among the Groups

Parameters	Group 1	Group 2	Group 3	P value		
				1-2	1-3	2-3
Exercise time (s)	396 ± 125	487 ± 66	519 ± 102	0.01	< 0.0001	NS
METs	7.61 ± 1.53	9.28 ± 1.86	9.87 ± 1.92	0.001	0.001	NS
SBP (mm Hg)	157 ± 18.22	143.12 ± 13.5	141.25 ± 13.6	0.011	0.01	NS
DBP (mm Hg)	88.5 ± 8.82	82.18 ± 3.63	82.08 ± 4.87	0.009	0.002	NS
HR (pulse/min)	148.4 ± 15.39	161.5 ± 10.99	161.5 ± 16.9	0.004	0.004	NS
E (m/s)	0.99 ± 0.16	0.97 ± 0.16	1.17 ± 0.16	NS	0.0001	0.001
A (m/s)	0.98 ± 0.21	0.94 ± 0.17	0.93 ± 0.16	NS	NS	NS
E/A	1.02 ± 0.16	1.02 ± 0.13	1.2 ± 0.17	NS	0.001	0.0001
DT (ms)	199.5 ± 23.5	193.8 ± 28.8	178.1 ± 21.2	0.48	0.001	0.05
IVRT (ms)	74.30 ± 8.60	69.7 ± 9.1	69.5 ± 7.4	0.009	0.03	NS
S (m/s)	0.65 ± 0.15	0.78 ± 0.13	0.84 ± 0.17	0.02	0.001	NS
D (m/s)	0.62 ± 0.15	0.68 ± 0.19	0.65 ± 0.12	NS	NS	NS
S/D	1.05 ± 0.27	1.2 ± 0.3	1.2 ± 0.2	NS	NS	NS
Ap (m/s)	0.45 ± 0.08	0.34 ± 0.07	0.32 ± 0.05	0.01	0.0001	NS
Em (m/s)	0.1 ± 0.07	0.13 ± 0.01	0.15 ± 0.03	NS	0.005	0.03
Am (m/s)	0.14 ± 0.09	0.12 ± 0.02	0.11 ± 0.02	NS	NS	NS
Em/Am	0.77 ± 0.23	1.0 ± 0.3	1.44 ± 0.5	0.011	0.0001	0.06
E/Em	11.1 ± 3.4	7.2 ± 1.4	7.7 ± 1.5	0.0001	0.0001	NS

SBP: systolic blood pressure; DBP: diastolic blood pressure; HR: heart rate; DT: deceleration time; IVRT: iso-volumetric relaxation time; MET: metabolic equivalent; NS: non-significant.

Ap wave velocity was higher in group 1 than the other two groups and the calculated value was over the normal limits. In group 1 lateral mitral annulus Em wave velocity and Em/Am ratio were lower than the other groups, while the E/Em ratio was detected significantly highest. The data of Doppler echocardiographical assessments and hemodynamic parameters after peak exercise among the groups are shown in Table 6. According to the findings, the exercise time and METs values were significantly low in group 1 when compared with groups 2 and 3 (7.61 ± 1.53 in group 1, 9.28 ± 1.86 in group 2 and 9.87 ± 1.92 in group 3, P value for group 1-2 = 0.001, group 1-3 = 0.001). Diastolic parameters in group 1 were changed and pseudo-normal pattern was seen after exercise; however, we could not find the similar changes in group 2 or 3. Similarly, Em/Am was significantly lower and E/E’ ratio was significantly increased in group 1.

## Discussion

Diastolic dysfunction, whose importance and the role in the pathophysiology of heart failure are being increasingly understood, has a high morbidity despite its low mortality rate [2, 3]. In a study conducted in patients admitted to hospital with a primary diagnosis of congestive heart failure, it was reported that 30-50% of all patients had preserved left ventricular systolic functions and the heart failure was caused by diastolic dysfunction [4]. The increased frequency of heart failure in patients with diabetes mellitus was demonstrated in both clinical and epidemiological studies [5]. Diastolic dysfunction is one of the first findings of this disease. In a study conducted in 206 patients by Bajraktari et al, diastolic dysfunction prevalence was found as high as 68% in the diabetic patients [6].

Doppler echocardiography is the most commonly used method for assessment of left ventricular diastolic function. Mitral and pulmonary vein Doppler analyses are being used for this purpose. Mitral annulus PWTD imaging is the relatively new approach for the assessment of left ventricular diastolic functions [7]. The data obtained from tissue Doppler imaging give more information about left ventricular filling pressures and survey.

In studies made on diastolic dysfunction, it was reported that this disorder was related to the decrease in the exercise performance [8-11]. In the study conducted by Poirier et al in 19 normotensive type 2 diabetic patients, they have revealed that patients with diastolic dysfunction have lower exercise performance than patients who have normal diastolic function [12]. Similarly in our study, exercise capacity was found lower in diabetic patients with diastolic dysfunction than similar patients in terms of age and sex whose diastolic functions were normal and than control individuals (exercise duration group 1: 396 ± 125 s, group 2: 487 ± 66 s, group 3: 519 ± 102 s).

In this study after peak exercise, a statistically significant increase in A wave velocity and a decrease in E/A ratio are detected in those with normal diastolic function and in those with diastolic dysfunction both E and A wave velocities are found increased. Furthermore, the increase in E wave velocity is statistically significant other than A wave increase. In patient with diastolic dysfunction E/A ratio increased and reached the normal value (E/A > 1). Impaired DT and IVRT decreased to normal limits.

Comparison of two diabetic groups revealed that patients with diastolic dysfunction showed significant D, S and Ap wave velocities after peak exercise while in patients without diastolic dysfunction, only the D wave velocity increased significantly. In the group with diastolic dysfunction, the increase in pulmonary vein reverse A wave velocity is significant and this value exceeded 0.35 m/s (Ap 0.35 ± 0.005 during relaxation, 0.45 ± 0.08 m/s; P = 0.002 during peak exercise).

Different from the other case studies which investigate the changes in diastolic function parameters after exercise in diabetic patients, our study reports mitral annular tissue Doppler velocities, as well. The tissue Doppler views of the all patients were taken before and after peak exercise. In the individuals with diabetic diastolic dysfunction, after peak exercise non-significant increases were observed in Em/Am ratio, in Em and Am wave velocities, while E/Em ratio increase was statistically significant. In diabetic patients whose diastolic functions were normal while there were significant increases in Em and Am velocities after exercise, Em/Am and E/Em ratios were similar to the rest values. In the control group, following the exercise while meaningful increases were observed in Em velocity and E/Em ratio was significantly changed. This finding may be explained by the limited amount of the study population.

E/Em ratio is one of the best echocardiographical parameters demonstrating the decrease in exercise capacities. In a study conducted by Skaluba et al in 121 patients, the relationship between left ventricular diastolic function parameters and duration of exercise was assessed and it was reported that the parameter showing the best correlation with the exercise capacity was E/Em ratio, decreased exercise tolerance was shown in patients with high E/Em ratios [13]. Similarly, in our study after peak exercise, the group with diabetic diastolic dysfunction showed lower exercise capacity and significantly higher E/Em ratio than the other groups. And this supports the relation between exercise capacity and E/Em ratio. The decrease of exercitional capacity suggests as a result of worsening diastolic functions with exercise when the increase in E/Em ratio is associated with high left ventricular filling pressure. This decrease in the duration of exercise is related to the worsening of diastolic functions during exercise. This worsening of diastolic functions arising from exercise has been demonstrated with the transformation of diastolic functions from abnormal relaxation pattern to peak at rest and to pseudo-normal left ventricular diastolic filling pattern after peak exercise.

Pseudo-normalization was demonstrated not only with the reversal of mitral E/A wave velocity ratio, but also with normalization of IVRT and pulmonary venous reverse flow velocity increase up to 0.35 m/s after peak exercise. Both this worsening in the left ventricular filling pattern and the increase in the E/Em ratio indicates that there are increases in left ventricular diastolic pressure and decrease in left ventricular relaxation rate after exercise. Depending on these, there would be increase in left atrium pressure as well. Due to worsening of abnormal left ventricular filling pattern, further increase in left atrium pressure may lead to increase in pulmonary vascular pressure and decrease in exercise tolerance. As a result of these changes, the duration of exercise would decrease. In a study conducted by Nair et al in 30 patients with hypertensive diastolic dysfunction, worsening in diastolic functions after exercise was found related to decrease in exercise performance [9].

The normal ventricle response to exercise is accelerated relaxation and associated with increased elastical relaxation. It is shown that both E and A velocities are increased with exercise. Relaxation disorder of myocardium during rest may not increase own relaxation under the hemodynamical stress of exercise and increased cardiac flow and may cause an increase in ventricular filling pressure. As a result, asymptomatic patients at rest may be symptomatic with a decrease in exercise duration [14]. Consequently, our study once more revealed that in patients with diabetic diastolic dysfunction exercise capacity decreases due to worsening of the existing diastolic dysfunction. Furthermore, this finding was supported by tissue Doppler echocardiographical inspection. We consider that further studies need to evaluate the efficacy of diastolic dysfunction treatment on the exercise capacity in diabetic patients with diastolic dysfunction.

The main limitation of the study is the amount of the study population. These findings might be academically stronger when the study consists of larger amount of patients. The re-evolution of the echocardiographic examination and exercise capacity after the treatment of diastolic dysfunction is another limitation of the study.

### Conclusion

Diabetic patients with diastolic dysfunction demonstrated a reduced exercise capacity, which may be due to aggravation of pre-existing left ventricular dysfunction.

## Abbreviations

A: late diastolic flow peak rate (with pulsed Doppler)
Am: late diastolic wave peak rate (with tissue Doppler)
Ap: pulmonary vein inverse flow rate
D: pulmonary vein diastolic flow peak rate
DT: deceleration time
E: early diastolic flow peak rate (pulsed Doppler)
Em: early diastolic wave peak rate (with tissue Doppler)
IVRT: isovolumetric relaxation time
PWTD: pulse wave tissue Doppler
S: pulmonary vein systolic flow peak rate
SPSS: Statistical Package for the Social Sciences
LVM: left ventricular mass
METs: metabolic equivalent
